# The effect of exposure to particulate matter during pregnancy on lower respiratory tract infection hospitalizations during first year of life

**DOI:** 10.1186/s12940-020-00645-3

**Published:** 2020-08-26

**Authors:** Sharon Goshen, Lena Novack, Offer Erez, Maayan Yitshak-Sade, Itai Kloog, Alexandra Shtein, Eilon Shany

**Affiliations:** 1 Department of Epidemiology, Faculty of Health Sciences, School of Medicine, Ben-Gurion University of the Negev, Beer Sheva, Israel; 2 Negev Environmental Health Research Institute, Soroka University Medical Center, Beer Sheva, Israel; 3 Department of Obstetrics and Gynecology, Faculty of Health Sciences, Soroka University Medical Center, School of Medicine, Ben-Gurion University of the Negev, Beer Sheva, Israel; 4 Exposure, Epidemiology, and Risk Program, Department of Environmental Health, Harvard T.H. Chan School of Public Health, Boston, MA USA; 5 Department of Geography and Environmental Development, Faculty of Humanities and Social Sciences, Ben-Gurion University of the Negev, Beer Sheva, Israel; 6 Department of Neonatology, Faculty of Health Sciences, Soroka University Medical Center, School of Medicine, Ben-Gurion University of the Negev, Beer Sheva, Israel

**Keywords:** Pneumonia, Bronchiolitis, Intrauterine exposure, PM_2.5_, Pregnancy, Hospitalizations

## Abstract

**Background:**

Lower respiratory tract infections (LRTI) in early life, including pneumonia, bronchitis and bronchiolitis, can lead to decreased lung function, persistent lung damage and increased susceptibility to various respiratory diseases such as asthma. In-utero exposure to particulate matter (PM) during pregnancy may disrupt biological mechanisms that regulate fetal growth, maturation and development. We aimed to estimate the association between intrauterine exposure to PM of size < 2.5 μm in diameter (PM_2.5_) and incidence of LRTIs during the first year of life.

**Methods:**

A retrospective population-based cohort study in a population of mothers and infants born in Soroka University Medical Center (SUMC) in the years 2004–2012. All infants < 1 year old that were hospitalized due to LRTIs were included. The main exposure assessment was based on a hybrid model incorporating daily satellite-based predictions at 1 km^2^ spatial resolution. Data from monitoring stations was used for imputation of main exposure and other pollutants. Levels of environmental exposures were assigned to subjects based on their residential addresses and averaged for each trimester. Analysis was conducted by a multivariable generalized estimating equation (GEE) Poisson regression. Data was analyzed separately for the two main ethnic groups in the region, Jewish and Arab-Bedouin.

**Results:**

The study cohort included 57,331 deliveries that met the inclusion criteria. Overall, 1871 hospitalizations of infants < 1 year old due to pneumonia or bronchiolitis were documented. In a multivariable analysis, intrauterine exposure to high levels of PM_2.5_ (> 24 μg/m^3^) in the first and second trimesters was found to be adversely associated with LRTIs in the Arab-Bedouin population (1st trimester, RR = 1.31, CI 95% 1.08–1.60; 2nd trimester: RR = 1.34, CI 95% 1.09–1.66).

**Conclusion:**

Intrauterine exposure to high levels of PM_2.5_ is associated with a higher risk of hospitalizations due to lower respiratory tract infections in Arab-Bedouin infants.

## Introduction

Respiratory diseases are a major cause of mortality and morbidity worldwide, particularly in infants and young children. Lower respiratory tract infections (LRTIs), including pneumonia and bronchiolitis, in early life may lead to a decreased lung function, and persistent lung damage, as well as increased susceptibility to various respiratory diseases like asthma later in life [1]. Risk factors for LRTIs are either intrinsic such as prematurity, congenital or acquired cardiopulmonary diseases, neuromuscular disorders and immunodeficiency [2–4] or external such as environmental crowding, exposure to cigarette smoke and postnatal exposure to air pollutants, such as particulate matter (PM) [5–7].

The Negev region in southern Israel is a part of the global dust belt extending from West Africa to the Arabian Desert, and is subjected to dust storms in which PM levels may increase by 10–100 fold [8]. The two major ethnic groups residing in this area are the Jewish and Arab-Bedouin, with the latter practicing a semi-nomadic way of living. The Jewish population lives in modern housing, usually works indoors and have higher socio-economic status. Approximately half of the Arab-Bedouin population lives in temporary settlements that cannot be hermetically sealed from outdoor pollutants and therefore is more likely to be exposed to and affected by them. These different lifestyles, coupled with the lower socioeconomic status create the difference between the two populations and has a potential to modify the environmental effects on health [9, 10].

Previous studies dealt with the association between postnatal exposure to PM and LRTIs [11–13]. Horne et al. found that short term exposure to PM_2.5_ is associated with LRTIs among infants younger than two years [14]. In our area, Yitshak-Sade et al. reported higher rates of hospitalizations due to bronchiolitis during or near elevated levels of PMs between 0 and 2 years of age [15].

Fetal cells that are in the process of differentiation have faster rate of replication and higher sensitivity to signals, and therefore, the fetus is likely to be more susceptible to environmental toxicants compared to adults [16–19]. Nevertheless, relatively few studies examined the effect of intrauterine exposure to pollution and early life respiratory infections [1, 20]. Some have found an association between prenatal exposure to PM and respiratory infections in children [1, 21, 22] while others did not find any association between the two, specifically for exposure to nitrogen dioxide (NO_2_) during pregnancy [23].

The lung maturation process takes a long period of time, extending from prenatal development through adolescence. In-utero development is composed of different phases that grossly correlate with the pregnancy trimesters [17, 24]. Studies investigating the prenatal effect of PMs on developing lungs in-vitro proposed different pathophysiological mechanisms: mice which were exposed to urban PMs showed elevated cytokine levels and increased levels of lipid and protein oxidation [25]. Likewise, in two studies prenatal and postnatal exposure to urban levels of PM_2.5_ were associated with decreased lung volumes [26, 27]. PM_2.5_ can cross the placenta and enter the bronchi and alveoli of the fetus compared to larger PMs [17, 28] and are associated with abnormal lung genesis and hyperactive lung disease [1, 20, 29].

In light of the unique climatic conditions and ethnic characteristics in the Negev area, we hypothesized that exposure to PM_2.5_ during gestation is independently associated with higher incidence of lung disease in children, and specifically lower respiratory tract infection in infants. The aim of our study was to assess the association of PM 2.5 exposure in the three trimesters of pregnancy and LRTIs in two different ethnic populations in southern Israel.

## Methods

We conducted a retrospective population-based cohort study of infants born in Soroka University Medical Center (SUMC) in southern Israel between the years 2004–2012, and their mothers. Included in this study were all women insured by Clalit Health Services (CHS), the largest health care provider in Israel. The follow-up of their infants was conducted until their first birthday. Only the first hospitalization due to LRTI was used in the analysis.

Women residing outside the exposure assessment area, i.e., the Negev region, women whose residence information was missing, and infants diagnosed with major congenital anomaly were excluded from the analysis.

Maternal demographic and clinical data on residential address, age at delivery, ethnicity, medical obstetrical history and birth outcomes were obtained from the Admission-Transfer-Delivery (ATD) database. Socioeconomic status was based on the evaluations by Israel Central Bureau of Statistics.

Due the known inherent difference between the two major ethnic groups in the region we a-priori intended to assess these groups separately.

### Exposure assessment

Exposure to PM_2.5_ was assessed based on a hybrid model developed by Kloog et al. [30, 31] utilizing daily satellite remote sensing data at 1km^2^ spatial resolution. The model uses an algorithm developed by the National Aeronautics and Space Administration (NASA) – called MAIAC (Multi-Angle Implementation to Atmospheric Correction) which is part of the MODIS (Moderate Resolution Imaging Spectroradiometer) system, providing satellite aerosol optical depth (AOD) data. The model then uses a hybrid LUR mixed model approach, by regressing daily PM_2.5_ mass concentration from MoEP (Ministry of Environmental Protection) monitoring networks against AOD, spatial predictors and temporal predictors. For days when AOD data are not available (because of meteorological conditions or retrieval errors) for some grid cells, the model fit a generalized additive model with a thin plate spline term of latitude and longitude to interpolate PM_2.5_. Model performance is excellent with out-of-sample cross validation R^2^ values of 0.92 and 0.87 for PM less than 10 μm in diameter (PM_10_) and PM_2.5_, respectively [31, 32].

In addition, we retrieved data on air pollution (PM_2.5_, PM_10_, NO_2_, sulphur dioxide (SO_2_), carbon monoxide (CO), ozone (O_3_)), temperature and relative humidity (RH) from 14 monitoring sites operated by the Ministry of Environmental Protection.

### Outcome definition

LRTI case was defined as the first hospitalization with pneumonia or bronchiolitis during the first year of life. The two morbidities were defined based on diagnoses recorded in the medical charts according to ICD9 codes 480–483, 486 and 466, for pneumonia or bronchiolitis, respectively. To minimize the contribution of postnatal exposure we limited the follow-up period to the first year of life.

### Statistical analysis

Quantitative variables were expressed as mean ± standard deviation, medians and ranges. Categorical variables were expressed by frequencies out of available cases. Hospitalization incidence was compared between sub-groups using t-test, Mann-Whitney test and Chi-Square or Fisher’s exact test.

Environmental exposures were assessed for collinearity and were found not highly correlated (Spearman rho< 0.8). Environmental exposures were averaged per each trimester, and presented as quartiles of pollutants concentrations, with the first quartile used as reference, to allow for a non-linear impact of the exposure on the outcome.

Prior to the multivariable analysis, all variables were examined in a univariable analysis for confounding and interactions in their association with the main exposure and the outcome at study. Only covariates that were identified as confounders and were significantly associated with both the exposure of interest and the outcome were included in the final models. The list of covariates included parity, preterm delivery, gender, low birth weight (< 2500 g), Apgar score at 5 min, ambient temperature, NO_2_ level, O_3_ level and cold season during first months of life.

For the assessment of associations between prenatal exposure to PM_2.5_ and LRTI-related hospitalization we used generalized estimating equation (GEE) models with Poisson distributed outcome while accounting for clusters of newborns born to the same mother during the study period. Relative risk (RR) represented the main measure of an effect. Models were corrected for overdispersion to meet the assumptions of the Poisson-based regression.

We also performed a sensitivity analysis, which included all confounders mentioned above and the mean exposure to PM_2.5_ during 1 year after birth (post-natal exposure).

Due to the exploratory nature of the study, we did not adjust to multiple comparisons in the analysis.

Statistical significance was set at *p*-value< 0.05. Data were analyzed using SPSS (IBM) software version 21.

## Results

During the study period there were 82,215 deliveries, of them 57,331 (70%) met the inclusion criteria and were included in the study. Women were excluded mainly due to missing exact address (19,111 women, 23.2%); residence out of the assessment area, or non CHS membership criterion (3760 women, 4.5%), deliveries of infants with major congenital malformations (1246 infants, 1.5%).

Among all women included in the analysis, the mean maternal age was 28.2 ± 5.7 years, and 40.5% of them were of Jewish ethnicity. In the newborn population, the mean gestational age was 38.9 ± 2 weeks, with 9.5% premature deliveries*.* Mean weight was 3148 g and 51% of them were males. The characteristics of the maternal and neonatal populations are summarized in Table 1 as a comparison between the Jewish and the Arab-Bedouin populations that demonstrate significant differences between the two groups. Arab-Bedouin women were on average 3 years younger at delivery, of lower socioeconomic status, more frequently resided in a rural area, were less likely to be diagnosed with diabetes during pregnancy and were less likely to be primiparous. In addition, their offspring were more frequently small-for-gestational age and they had a higher proportion of infants born with lower Apgar score. Maternal and neonatal characteristics by LRTI incidence are presented in Table 2.

**Table 1:** Maternal and neonatal characteristics by Jewish and Arab-Bedouin origin

	Jewish origin (***n*** = 23,254)	Arab-Bedouin origin (***n*** = 34,077)	p-value
*Demographic characteristics*			
Age, years			
Mean ± SD, (n)	30.0 ± 5.0 (23,243)	27.0 ± 6.0 (34,063)	< 0.001
Median	29.0	27.0	
Low tertile of socioeconomic status, % (n)	2.1 (499)	66.6 (22,692)	< 0.001
Address, % (n),			
Urban	92.2 (21,443)	78.3 (26,185)	< 0.001
Rural	3.0 (566)	6.5 (2250)	
*Medical and obstetrics history*			
Gestational Diabetes Mellitus, % (n)	4.7 (1083)	2.7 (911)	< 0.001
Primiparous, %, (n)	31.8 (7383)	19.3 (6574)	< 0.001
*Neonatal data*			
Gestational age (weeks)			
Mean ± SD, (n)	38.8 ± 2.0 (23,223)	39.1 ± 2.1 (34,045)	< 0.001
Median	39.0	39.3	
Preterm delivery, % (n)	9.7 (2248)	9.5 (3249)	0.585
Male gender, % (n)	50.9 (11,827)	51.1 (17,427)	0.508
Birth weight, gram			0.335
Mean ± SD, (n)	3151 ± 541 (23,237)	3147 ± 541 (34,057)	
Median	3180	3168	
Small for Gestational Age (SGA), % (n)	3.4 (774)	4.4 (1500)	< 0.001
Large for Gestational Age (LGA), % (n)	6.7 (1531)	6.8 (2283)	0.585
Apgar score			
1st minute < 5, % (n)	5.3 (1218)	7.1 (2368)	< 0.001
5th minute < 7, % (n)	0.6 (149)	1.0 (333)	< 0.001

**Table 2:** Maternal and neonatal characteristics by LRTI incidence

	Jewish origin			Arab-Bedouin origin		
	Without LRTI (***n*** = 22,724)	LRTI (***n*** = 530)	p-value	Without LRTI (***n*** = 32,736)	LRTI (***n*** = 1341)	p-value
*Demographic characteristics*						
Age, years						
Mean ± SD, (n)	30 ± 5 (22,724)	29 ± 5 (530)	0.203	27 ± 6 (32,736)	28 ± 6 (1341)	0.04
Median	29	29		27	27	
Low tertile of socioeconomic status, % (n)	2.3 (485)	2.8 (14)	0.02	66.4 (21,762)	69.3 (930)	0.05
*Medical and obstetrics history*						
Gestational Diabetes Mellitus, % (n)	4.7 (1058)	4.7 (25)	0.94	2.6 (859)	3.9 (52)	0.005
Primiparous, % (n)	32.1 (7290)	17.6 (93)	< 0.001	19.6 (6419)	11.6 (155)	< 0.001
*Neonatal data*						
Gestational age (weeks),						
Mean ± SD, (n)	38.8 ± 2 (22,724)	38.2 ± 2 (530)	< 0.001	39.1 ± 2 (32,736)	38.5 ± 2 (1341)	< 0.001
Median	39	38.8		39.2	39	
Preterm delivery, % (n)	9.5 (2159)	16.8 (89)	< 0.001	9.2 (3025)	16.7 (224)	< 0.001
Male gender, % (n)	50.7 (11,527)	56.6 (300)	0.007	51 (16,698)	54.4 (729)	0.01
Birth weight, gram						
Mean ± SD, (n)	3154 ± 539 (22,724)	3062 ± 611 (530)	< 0.001	3151 ± 537 (32,736)	3045 ± 625 (1341)	< 0.001
Median	3180	3105		3180	3110	
Small for Gestational Age (SGA), % (n)	3.3 (756)	2.8 (18)	0.90	4.4 (1440)	4.5 (60)	0.92
Large for Gestational Age (LGA), % (n)	6.7 (1501)	5.8 (30)	0.406	6.8 (2191)	6.9 (92)	0.84
Apgar score						
1st minute < 5, % (n)	5.3 (1185)	6.3 (33)	0.312	6.9 (2234)	10.2 (134)	< 0.001
5th minute < 7, % (n)	0.6 (144)	0.9 (5)	0.256	0.9 (304)	2.2 (29)	< 0.001

During the study period, 2079 LRTI-related hospitalizations were recorded in the cohort of infants < 1 year old: 784 were due to pneumonia and 1295 were due to bronchiolitis, overall 1871 primary hospitalizations after excluding 208 repeated hospitalizations.

We detected higher rates of hospitalizations due to LRTIs, and separately due to pneumonia or bronchiolitis within the Arab-Bedouin population (LRTI: 3.9% vs. 2.3%, *p* < 0.001) as compared to infants born to Jewish mothers. The rate of hospitalized infants which had the highest in utero exposure to PM_2.5_ (Q_4_) was also higher in the Arab-Bedouin population (5% vs. 2.5%, p < 0.001).

The environmental factors are presented in Table 3. The mean of pollutants rate was higher in the Jewish population. Specifically, the average daily PM_2.5_ exposure over the entire pregnancy in Jewish population was 22.3 ± 2.1 μg/m^3^, with an IQR of 20.6–23.7 μg/m^3^ (Q_1_: 13–20.6 μg/m^3^; Q_2_: 20.6–22.0 μg/m^3^; Q_3_: 22.0–23.7 μg/m^3^; Q_4_: 23.7–60.4 μg/m^3^). The average daily PM_2.5_ exposure over the entire pregnancy in Arab-Bedouin population was 22.0 ± 1.7 μg/m^3^, with an IQR of 20.7–23.1 μg/m^3^ (Q_1_: 13.5–20.7 μg/m^3^; Q_2_: 20.7–21.7 μg/m^3^; Q_3_: 21.7–23.1 μg/m^3^; Q_4_: 23.1–77.0 μg/m^3^).

**Table 3:** Environmental factors in pregnancy in the study population

Environemntal factors	Jewish origin (n = 23,254)			Arab-Bedouin origin (n = 34,077)			p-value^**1**^
	Min-Max	Mean ± SD Median	Inter-Quartile Range (IQR)	Min-Max	Mean ± SD Median	Inter-Quartile Range (IQR)	
PM_2.5_, μg/m^3^	13.0–60.4	22.3 ± 2.1 22.0	20.6–23.7	13.5–77.0	22.0 ± 1.7 21.7	20.7–23.1	< 0.001
PM_10_, μg/m^3^	14.7–185.1	53.1 ± 6.5 52.9	48.2–57.1	23.2–168.5	50.2 ± 6.2 49.7	45.6–54.3	< 0.001
NO_2_, ppb	1.3–22.9	9.6 ± 1.7 9.9	8.8–10.8	1.30–24.10	9.5 ± 1.7 9.8	8.8–10.6	< 0.001
SO_2_, ppb	0.02–4.37	1.7 ± 0.4 1.8	1.6–2.0	0.01–3.62	1.6 ± 0.5 1.8	1.3–2.0	< 0.001
O_3_, ppb	10.45–55.83	36.5 ± 3.0 36.5	34.8–38.6	6.85–56.79	36.3 ± 4.0 36.5	34.7–38.6	< 0.001
Temp, **°**C	9.17–32.19	20.1 ± 1.8 20.1	18.6–21.5	8.7–29.11	20.0 ± 1.7 20.1	18.6–21.4	< 0.001
Humidity, %	16.65–86.8	60.4 ± 15.5 65.4	59.0–67.6	16.44–83.02	60.4 ± 15.7 65.4	59.0–67.8	0.960

^1^Data evaluated with t-test or Mann-Whitney test

**Fig. 1:**
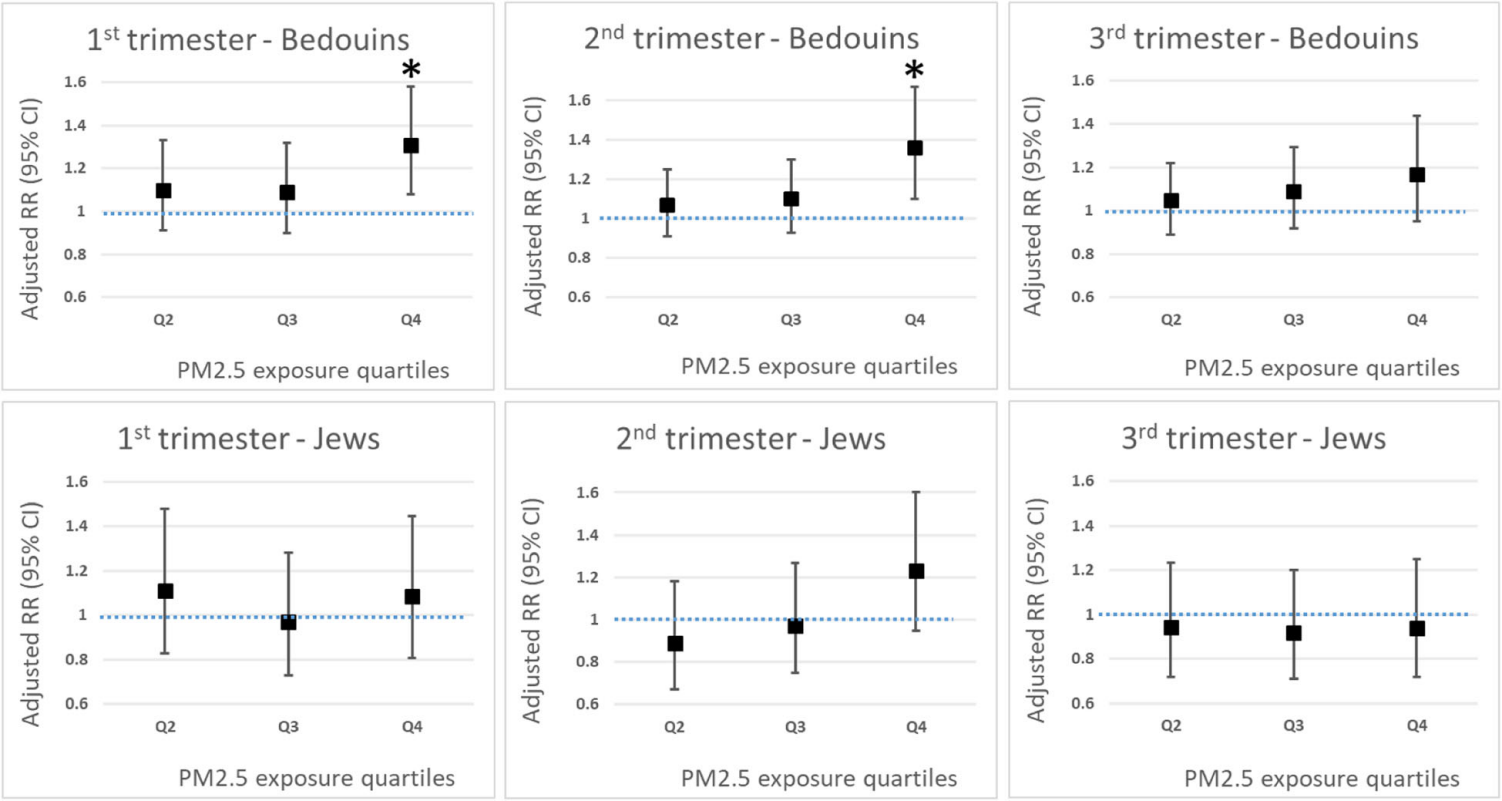
Association between intrauterine exposure to PM2.5 and LRTI by quartiles, based on a multivariable analysis^1. 1^Associations were adjusted for gender of a newborn, parity, preterm delivery, birth weight and Apgar score at 5 min, temperature, NO_2_, Ozone level and cold season during first months of life. Environmental exposure was averaged per each trimester, and presented as quartiles of pollutants’ concentrations, with the first quartile used as reference. * *p* < 0.05 for comparison with the first quartile of PM_2.5_ exposure

After adjustments to relevant demographical, clinical and environmental factors (see Methods), highest exposure (4th quartile) to PM_2.5_ in the first and second trimesters was found to be adversely associated with LRTI-related hospitalizations in the subgroup of the Arab-Bedouin population (RR = 1.31; 95% CI 1.08–1.60 and RR = 1.34; 95% CI 1.09–1.66 for the first and second trimesters, respectively, Fig. 1). We did not find statistically meaningful associations in the Jewish population.

Sensitivity analysis models, which included postnatal exposure to PM_2.5_ did not change the results **(**RR = 1.26; 95% CI 1.02–1.54 and RR = 1.34; 95% CI 1.07–1.68 for the exposure to the 4th quartile of PM_2.5_ averaged over the first and second trimesters in the Arab-Bedouin population, respectively).

## Discussion

In our current study, we examined an effect of intrauterine exposure to pollutants on the risk of LRTI. We found that exposure to higher levels of PM_2.5_ in-utero is associated with a higher risk of LRTI-related hospitalizations in the Arab-Bedouin sub-population of the Negev area. This effect was not demonstrated within the Jewish population.

Based on previous research, the association demonstrated above may be explained by several mechanisms. Generally, chronic exposure to external factors during pregnancy, such as PM, may disrupt biological mechanisms that regulate fetal growth, maturation and development [1, 16, 17, 33]. Specifically, exposure to PMs during pregnancy may induce oxidative stress that cause inefficient repair mechanisms of the developing lung [17, 24, 34] and genetic modification that probably contribute to an increased pulmonary susceptibility [1, 20, 35–37]. In addition to classical genetic mechanisms, environmental stimuli may induce acquired epigenetic states that affect gene expression and phenotypic outcome [38, 39].

Several investigations have already assessed the adverse effect of prenatal exposure to PM_2.5_ and asthma disease [20, 40]. Although different pathophysiology (as asthma is an inflammatory disease and not infectious), oxidative stress seems to play a role in both diseases and eventually contribute to more vulnerable infants [41]. In addition, researchers in previous studies have reported on the second trimester as the sensitive window to asthma disease [29, 42]. Our results partially support this observation, as we find the beginning and mid gestation periods to be the vulnerability window most relevant for environmental exposures.

In our study, exposure to PM_2.5_ was found to be associated with health outcome only in the Arab-Bedouin population and not among the Jewish population. The discrepancy in findings between the two sub-groups is intriguing and may have several possible explanations:

First, the Jewish population is attending the community medical services more often, and are more compliant with preventive medicine lifestyle [43], thus it is possible that their respiratory infections are treated earlier and do not require hospitalization, as opposed to patients of Arab-Bedouin origin. Secondly, as demonstrated in Table 1, the Arab-Bedouin population has different characteristics compared to the Jewish population, that has to do with many aspects of life, which may support the assumption that their actual exposure to outdoor pollutants is higher. Likewise, as shown in other studies, this population is more likely to be exposed to cigarette smoke and open fire cooking, conditions that may increase the effect of prenatal exposure to other pollutants [44, 45]. This behaviour is strongly correlated with a low socio-economic status, and hence, poverty, that as been shown, enhances the effects of all risk factors, and specifically of environmental risk factors [46–48]. In our case, the aforementioned risky behaviours may lead to an increased risk for LRTIs within Arab-Bedouin infants.

Our study had several limitations. As most of the rural Arab-Bedouin settlements do not have a permanent address (they use a P.O. Box instead) they were excluded from the study analysis and a possible selection bias might have ensued. However, since the excluded population was probably more likely to be exposed to higher PM levels, we probably underestimated the effect of PM_2.5_. In addition, the exposure estimation in our analysis is linked to the women’s residential address at delivery hospitalization. As the information on changing residence was not available to the researchers, which might have resulted in a minor information bias in exposure assessment.

Another limitation is related to indoor exposure to pollutants and/or the accurate time pregnant women were outdoors in their residence area, data that we were unable to collect and may have provided more accurate estimates of exposure. As a result, this study is prone to a non-differential misclassification in exposure assessment and consequently, a decreased statistical power of the study. However, it is most probably driving the study towards the null hypothesis.

Finally, information about maternal smoking or postnatal environmental tobacco exposure were severely underreported and therefore not used in multivariable analysis. Since perinatal and postnatal exposure to tobacco smoke has been found to be associated with infant’s respiratory morbidity, this lack of information may bias our results. However, previous studies that assessed the quantity of tracers of cigarettes smoking (cadmium and cotinine) in pregnant Arab women, found that they appear the least exposed to passive smoking, and that these tracers had very low concentrations in this population [9, 49]. Likewise, the information on open fire cooking rates in the Arab-Bedouin population was not available from medical databases and therefore could not be included in the analysis. As a result, the study conclusions may be prone to a certain residual confounding, not fully accounted in spite of the extensive adjustment performed in the models.

The current investigation benefited from the large sample size, which allowed an efficient adjustment of the estimates to multiple co-variates. Furthermore, this is a population-based study, where all cases were hospitalized in the single hospital in the Negev region ensuring an accurate case ascertainment of all severe LRTI cases in the area. We also used an exposure model with a high spatial resolution, permitting an estimation of exposure levels to PMs with high accuracy.

## Conclusions

In this large population-based study, higher prenatal exposure to PM_2.5_ was found to be adversely associated with LRTIs during the first year of life in the Arab-Bedouin population in southern Israel. Our results add to the accumulating data on the harmful effect of PMs on human health, while focusing on one of the most vulnerable periods of life. Further studies are necessary to ascertain the relevant window of exposure and the most harmful pollutants in order to devise effective measures to deal with these problems.

## Data Availability

The datasets during and/or analyzed during the current study available from the corresponding author on reasonable request.
